# Draft Genome Sequence, and a Sequence-Defined Genetic Linkage Map of the Legume Crop Species *Lupinus angustifolius* L

**DOI:** 10.1371/journal.pone.0064799

**Published:** 2013-05-29

**Authors:** Huaan Yang, Ye Tao, Zequn Zheng, Qisen Zhang, Gaofeng Zhou, Mark W. Sweetingham, John G. Howieson, Chengdao Li

**Affiliations:** 1 Department of Agriculture and Food Western Australia, South Perth, Australia; 2 Beijing Genome Institute, Shenzhen, China; 3 Centre for Rhizobium Studies, Murdoch University, Murdoch, Australia; University of California, Riverside, United States of America

## Abstract

Lupin (*Lupinus angustifolius* L.) is the most recently domesticated crop in major agricultural cultivation. Its seeds are high in protein and dietary fibre, but low in oil and starch. Medical and dietetic studies have shown that consuming lupin-enriched food has significant health benefits. We report the draft assembly from a whole genome shotgun sequencing dataset for this legume species with 26.9x coverage of the genome, which is predicted to contain 57,807 genes. Analysis of the annotated genes with metabolic pathways provided a partial understanding of some key features of lupin, such as the amino acid profile of storage proteins in seeds. Furthermore, we applied the NGS-based RAD-sequencing technology to obtain 8,244 sequence-defined markers for anchoring the genomic sequences. A total of 4,214 scaffolds from the genome sequence assembly were aligned into the genetic map. The combination of the draft assembly and a sequence-defined genetic map made it possible to locate and study functional genes of agronomic interest. The identification of co-segregating SNP markers, scaffold sequences and gene annotation facilitated the identification of a candidate R gene associated with resistance to the major lupin disease anthracnose. We demonstrated that the combination of medium-depth genome sequencing and a high-density genetic linkage map by application of NGS technology is a cost-effective approach to generating genome sequence data and a large number of molecular markers to study the genomics, genetics and functional genes of lupin, and to apply them to molecular plant breeding. This strategy does not require prior genome knowledge, which potentiates its application to a wide range of non-model species.

## Introduction

Wild types of narrow-leafed lupin (*Lupinus angustifolius* L.) were grown in classical Greek and Roman times [1]. These genotypes were bitter (with the seed containing approximately 1.5% alkaloids), hard-seeded (seeds were impermeable to water and remained dormant after sowing), pods shattered at maturity, and were late-flowering. The first step in modern lupin breeding began in Europe with the selection of a natural, low alkaloid mutant by von Sengbusch [2]. Domestication of this plant species was completed in the 1960’s in Western Australia. The first fully domesticated cultivar (with low alkaloids, non-shattering pods, permeable seeds and early flowering) was Unicrop, released in 1973. Domesticated *L. angustifolius*, often called as “Australian sweet lupin”, is now a major grain legume crop in southern Australia, and is also cultivated in Europe, America and South Africa [3]. Seeds of the Australian sweet lupin are high in protein (30–35%) and dietary fiber (30%), but are low in oil (6%), and contain negligible starch [4], [5]. Sweet lupin has the lowest glycemic index (GI) among commonly consumed grains (http://www.lupins.org). Medical and dietetic studies have shown that consuming sweet lupin-enriched food has significant health benefits, including suppression of appetite and energy intake [6], [7], reduced blood glucose and insulin response [8], improved blood lipids [9], and improved bowel health indicators [10]. Apart from being a profitable crop itself, the cultivation of lupin benefits cereal crops grown in rotation with it, because of nitrogen fixation through rhizobium nodulation [11], and from control of soil-borne root diseases.

*L. angustifolius* is a diploid plant species containing (2 n) 20 pairs of chromosomes [1]. In the last 10 years, the DNA fingerprinting method of microsatellite-anchored fragment length polymorphism (MFLP) [12] has been applied to molecular marker development in lupin, from which we have established DNA markers linked to a number of key disease resistance genes and domestication genes. Many of these markers have been applied in marker-assisted selection (MAS) in the Australian lupin breeding program [13]–[23]. Unfortunately, all these markers were of small DNA size (under 500 bp each), which provide little value for interpreting or exploiting the lupin genome. Several genetic linkage maps have been reported for this species [24]–[26]. However, less than 400 markers on these maps have DNA sequence information, and the majority of the markers in previous maps were anonymous [26]. It is highly desirable to construct a sequence-defined genetic map which can be unambiguously transferred and interpreted among all lupin breeding germplasm, and be applicable to comparative genomics studies for other plant species. Naganowska *et al*. [27] reported that the C-value of the nuclear DNA content [28] of *L. angustifolius* was 1.89 pg. At present, there is little other published knowledge of the lupin genome, aside from a small portion of a bacterial artificial chromosome (BAC) library which was end-sequenced [29].

Cultivar Tanjil was released in Australia in 1998. It became the dominant cultivar in the early 2000’s because it is highly resistant to the disease anthracnose, caused by the fungal pathogen *Colletotrichum lupini*. This is the most devastating disease of lupin [30]. Tanjil is also high-yielding, resistant to the disease phomopsis stem blight (caused by fungal pathogen *Diaporthe toxica*), grey leaf spot disease (caused by *Stemphylium botryosum*), CMV virus transmission, and aphid colonization. Because of these favorable characters, Tanjil has been extensively used as a parental line in subsequent crossing, and is thus associated with a wide range of elite germplasm in lupin breeding programs. Study of the genome sequence and genetics of Tanjil is therefore highly relevant to ongoing lupin breeding. Here, we report the draft genome assembly from a whole genome shotgun sequencing dataset of *L. angustifolius* obtained from Tanjil. We also report integration of the genome sequence data, sequence-defined DNA markers and metabolic pathways as an efficient approach to identifying genes associated with economically important traits.

## Results

### Genome Sequencing and Gene Annotation

We obtained 31.001 billion base pairs (bp) of high quality sequencing data. With the 17-mer analysis model [31], the peak of the 17-mer distributed at 22, and the K-mer frequencies along the sequencing depth gradient followed a Poisson distribution. The total K-mer count was 25,376,847,185. Based on the G = K-number/peak depth model [31], the lupin genome size was estimated at 1.153 Gb. Thus, the genome sequencing data represented 26.9x coverage of the lupin genome.

The repeat content was estimated by the 17-mer production cumulative curve. The production cumulate concentrated at high depth kmer (>44x), while the low depth kmer (depth <44x) was at 26.53%. The repeat sequence content in the lupin genome was estimated at 50% based on the K-mer plot model [31], [32]. The percentage of repeat sequences in lupin is comparable to those in other legume species such as soybean (59%) [33], castor bean (50%) [34], and pigeonpea (52%) [35].

The draft genome assembly was constructed using the software program SOAPdenovo [31]. The total length span of assembled scaffolds was 598 Mbp, which was approximately 51.9% of the total genome size (Table 1). The number of scaffolds >2 kb was 51,867 with the total length span at 538 Mbp, which accounted for 90% of the genome sequence assembly (Table 1). The scaffolds of the lupin draft genome assembly have been deposited at the Genbank (Submission number “SUB139069”, project number “PRJNA179231”).

**Table 1:** Genome sequence assembly and annotation statistics for *Lupinus angustifolius.*

	All scaffold	Scaffolds longer than 2 kb
Number of scaffolds	234,534	51,867
Total span	598 Mb	538 Mb
Scaffold N50	12.546 kb	15.597 kb
Longest scaffold	151.003 kb	151.003 kb
Average scaffold length	2.511 kp	10.372 kp
Longest contig	70.567 kb	70.567 kb
Average contig length	927 bp	6,470 bp
Number of contigs	457,917	59,292
Contig N50	5,806 bp	
GC content	33.61%	
Gene models	57,806	
Mean transcript length	2,038.6 bp	
Mean coding sequence length	1,033.5 bp	
Mean number of exons per gene	4.09	
Mean exon length	252 bp	
GC content in exons	41.32%	
Mean intron length	325 bp	
GC content in introns	32.11%	

Annotation of the lupin genome sequences resulted in the identification of 57,807 genes, with the average transcript length at 2,038 bp, and coding size at 1,033 bp. The average lengths of exon and intron were 252 bp and 325 bp, respectively. Each gene consisted of average 4.09 exons (Table 1). The annotated 57,807 genes and their positions on the respective scaffolds are presented in the annotation dataset in Table S1. The number of genes identified in lupin (57,807) was greater than those found in other legume species such as *Lotus japonicas* (38,483) [36], *Medicago truncatula* (47,529) [35], *Glycine max* (46,430) [33], and *Cajanus cajan* (48,680) [35]. This might reflect the fact that the size of lupin genome (1.153 Gb) is larger than that of *Medicago truncatula* (475 Mbp) [37], *Lotus japonicas* (472 Mbp) [36], *Cajanus cajan* (833 Mbp) [35], and *Glycine max* (950 Mbp) [33].

### Construction of a Sequence-defined Genetic Map

**Figure 1:**
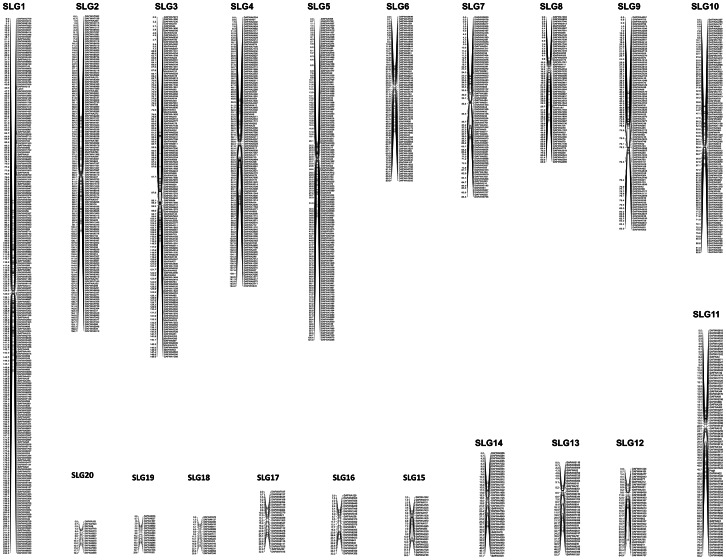
A framework linkage map of the *Lupinus angustifolius* genome. The framework map is extracted from the full map which consisted of 8,246 sequence-defined molecular markers. Detailed lists of all the molecular markers in the sequence-defined lupin map, including their genetic distance in each linkage group, the DNA sequences, and their corresponding scaffolds in the genome sequence assembly, are presented in Table S2.

The restriction-site associated DNA sequencing (RAD-seq) analysis on the two parental plants Unicrop and Tanjil and their resultant 94 F_8_ recombinant inbred lines (RILs) produced 8,244 sequence-defined markers (Table 2), including 7,563 SNP markers and 681 indel (insertion/deletion) markers (Table S2). Linkage analysis placed these SNP/indel markers and the two previously developed sequence-defined markers AntjM1 and AntjM2 linked to the anthracnose disease resistance gene *Lanr1* [13], [38] into 20 linkage groups (Table 2). A detailed genetic linkage map containing 8,246 sequence-defined markers, including the DNA sequences and the genetic distance within each sequence-defined linkage group (SLG), is presented in the mapping dataset in Table S2. The linkage groups were designated as SLGs to differentiate them from previous LGs (linkage groups) which were based predominantly on anonymous markers [24]–[26]. The total length of the linkage map was 1,629.9 centiMorgans (cM) (Table 2), which is similar to the length reported in *L. angustifolius* by Boersma *et al.* [24]. The average density of this map was at 5.1 markers per cM (Table 2). A framework genetic linkage map showing the 1,517 loci extracted from the full map is presented in Figure 1.

**Table 2:** Summary of the genetic linkage map constructed based on 8,246 sequence-defined molecular markers in *Lupinus angustifolius.*

Linkage groups	Number of sequence-defined markers	Number of loci	Genetic length (cM)	Number of integrated scaffolds	Scaffold length span (bp)
SLG-1	1498	235	234.3	763	129,562,88
SLG-2	1496	140	156.7	724	11,025,395
SLG-3	414	151	149.0	236	4,368,678
SLG-4	814	120	144.2	400	6,428,848
SLG-5	785	143	101.9	365	6,243,667
SLG-6	242	73	89.0	129	2,743,736
SLG-7	218	81	86.5	114	2,326,261
SLG-8	539	64	85.0	289	4,760,577
SLG-9	299	95	83.5	155	2,970,440
SLG-10	232	104	82.6	138	2,151,365
SLG-11	690	85	82.2	344	6,059,460
SLG-12	246	34	64.9	143	2,103,598
SLG-13	266	36	52.2	155	2,321,041
SLG-14	102	40	51.1	57	1,150,524
SLG-15	69	23	34.5	32	631,989
SLG-16	81	23	33.3	47	733,299
SLG-17	82	24	32.4	40	766,926
SLG-18	68	16	26.6	28	733,186
SLG-19	23	16	20.6	13	572,743
SLG-20	82	14	19.4	42	703,582
Sub total	8,246	1,517	1629.9	4,214	71,751,603

### Integration of Assembled Scaffolds into the Linkage Map

Blast searching using DNA sequences of the SNP/indel markers against the draft assembly assigned a total of 4,214 scaffolds into the sequence-defined lupin genetic map (Table 2, Table S2). The total DNA length span of the scaffolds integrated into the map was 71,751,603 bp, which represented 12% of the lupin genome sequence assembly (Table 2), or 6.2% of the lupin genome size. The results of gene annotation on the 4,214 scaffolds integrated on the sequence-defined genetic linkage map are presented in Table S3.

### Identification of a Candidate Gene for Anthracnose Disease Resistance

Selection for anthracnose resistance is one of the key objectives in lupin breeding programs. In the last 10 years, several molecular markers have been developed tagging the R gene *Lanr1* for MAS, and the genetic distance has been gradually improved from 3.5 cM [14], to 2.3 cM [38], and more recently to 0.9 cM [39]. In the present study, 37 sequence-defined SNP markers (the markers which are highlighted in green in Table S2) were found to be linked to the R gene *Lanr1* conferring anthracnose disease resistance [14] within the genetic distance of 5 cM. Two of these markers, DAFWA213 and DAFWA5820, were co-segregating (0 cM) with the R gene *Lanr1* among the 94 RILs (Table S2). These two SNP markers were mapped on linkage group SLG1 (Figure 1, Table S2). Both markers were located on the same scaffold 31581, which is 15,706 bp in length (Table S2). Gene annotation analyses showed that the scaffold 31581 encoded a TIR-NBS-LRR type protein (Figure S1), which is a typical structure of plant disease resistance genes [40]. Therefore, the TIR-NBS-LRR gene on scaffold 31581 is considered as a candidate R gene associated with anthracnose resistance in lupin. Sequence analysis by software of Conserved Domain Database (CDD) [41] on this candidate R gene has detected several conserved domains, including TIR, P-loop (GTGKTT), NB-ARC, kinase-2 (LLVLDD), GLPLAL, and MHD (Figure S1); all these are typical domains found in many plant disease resistance genes [40], [42]. We further tested the SNP marker DAFWA213 on a larger segregating population containing 190 F_8_ RILs which resulted from the cross Unicrop x Tanjil. All of these 190 RILs showed complete consistency between marker genotypes and anthracnose disease phenotypes, which further confirmed the association between the R gene *Lanr1* and marker DAFWA312 and its corresponding scaffold 31581. Furthermore, we tested the SNP marker DAFWA213 on Australian historical and current commercial lupin cultivars for marker validation, which confirmed that the marker genotypes were consistent with the disease resistance genotypes on all these cultivars (Table S4), indicating their close association with the target gene [43], [44]. This evidence, when combined, strongly indicated that the TIR-NBS-LRR gene in scaffold 31581 was the candidate gene for anthracnose disease resistance, although more research is required to confirm the relationship between gene function and the expression of anthracnose disease resistance in lupin.

### Identification of Scaffolds for Molecular Markers Linked to Genes of Agronomic Traits of Interest

The parental plant Tanjil has dark speckles on the seed coat, while the parental line Unicrop is white. The F_8_ population used in map construction in this study segregated for seed coat colour. Genetic linkage analysis found that 63 markers (highlighted in yellow in Table S2) were linked to the seed coat colour within 5 cM. Twenty four markers were co-segregating (0 cM) with the seed coat colour gene (highlighted in pink in Table S3). A blast search of the lupin genome assembly found 16 scaffolds bearing SNP markers co-segregating with seed coat colour (Table S2; Table 3). The seed coat colour gene and its associated markers are mapped on the linkage group SLG8 (Figure 1). No gene sequences homologous with known plant pigment genes were identified from sequence analysis on the 24 co-segregating SNP/indel markers and the 16 co-segregating scaffolds.

**Table 3:** Identification of scaffolds containing molecular markers linked to key agronomic genes in *Lupinus angustifolius.*

Agronomic traits	Name of markers	Distance between marker and target gene (cM)	Reference source	Scaffold identified	Scaffold size (bp)
Disease resistance gene *Lanr1*	*DAFWA213*	0	This study	scaffold 31581	15,706
Disease resistance gene *Lanr1*	*DAFWA5820*	0	This study	scaffold 31581	15,706
Disease resistance gene *Lanr1*	AntjM1	3.5	[14]	scaffold83350	11,407
Disease resistance gene *Lanr1*	AntjM2	2.3	[38]	scaffold2992	33,979
Disease resistance gene *Lanr1*	AnSeq3	0.9	[39]	Scaffold33942	64,039
Disease resistance gene *Lanr1*	AnSeq4	0.9	[39]	Scaffold31346	33,727
Disease resistance gene *PhtjR*	**DAFWA4522**	0	This study	Scaffold98007	17,744
Disease resistance gene *PhtjR*	**DAFWA7825**	0	This study	Scaffold98007	17,744
Disease resistance gene *PhtjR*	**DAFWA6895**	0	This study	Scaffold84773	33,448
Disease resistance gene *PhtjR*	**DAFWA4020**	0	This study	Scaffold84773	33,448
Disease resistance gene *PhtjR*	**DAFWA3123**	0	This study	Scaffold84773	33,448
Seed coat colour	***DAFWA7513***	0	This study	C28631230	1,673
Seed coat colour	***DAFWA2297***	0	This study	scaffold10511	21,570
Seed coat colour	***DAFWA4071***	0	This study	scaffold11205	12,869
Seed coat colour	***DAFWA6428***	0	This study	scaffold11676	22,481
Seed coat colour	***DAFWA4544***	0	This study	scaffold13708	44,176
Seed coat colour	***DAFWA8063***	0	This study	scaffold18757	3,783
Seed coat colour	***DAFWA4022***	0	This study	scaffold18978	10,158
Seed coat colour	***DAFWA8668***	0	This study	scaffold19305	14,364
Seed coat colour	***DAFWA1978***	0	This study	scaffold23047	5,252
Seed coat colour	***DAFWA1986***	0	This study	scaffold30742	10,173
Seed coat colour	***DAFWA2406***	0	This study	scaffold35025	4,343
Seed coat colour	***DAFWA805***	0	This study	scaffold7657	3,023
Seed coat colour	***DAFWA2968***	0	This study	scaffold87947	4,118
Seed coat colour	***DAFWA2038***	0	This study	scaffold13764	19,254
Seed coat colour	***DAFWA7504***	0	This study	scaffold40404	6,621
Seed coat colour	***DAFWA939***	0	This study	Scaffold48877	15,086
Disease resistance gene *AnMan*	AnManM1	5.1	[15]	scaffold36514	50,220
Disease resistance gene *Phr1*	Ph258M1	5.7	[13]	scaffold84752	21,471
Disease resistance gene *Phr1*	Ph258M2	2.1	[13]	scaffold16252	15,559
Resistance gene against lupin rust disease	RustM1	unknown	Yang et al unpublished data	scaffold15347	42,210
Early flowering gene *Ku*	KuH	0	[18]	scaffold21489	30,923
Soft-seed coat gene *mollis*	MoA	0	[16]	scaffold75616	14,783
Soft-seed coat gene *mollis*	MoLi	0	[23]	scaffold75616	14,783
Pod-non-shattering *le*	LeLi	6.0	[22]	scaffold87978	9,909
Pod-non-shattering gene *le*	LeM2	1.3	[17]	scaffold79908	20,738
Pod-non-shattering gene *tardus*	TaM1	2.1	[19]	scaffold15347	21,529
Pod-non-shattering gene *tardus*	TaLi	1.4	[20]	scaffold36274	8,191
Low alkaloid gene *iucundus*	IucLi	0.9	[21]	scaffold30160	20,677

Markers co-segregating (0 cM) with the *Lanr1* gene conferring resistance to anthracnose disease are highlighted in italics; markers co-segregating with the *PhtjR* gene conferring resistance against phomopsis stem blight are highlighted in bold; and markers co-segregating with the seed coat colour gene are highlighted in italics and bold. These highlighted markers were developed in this study. The remaining 16 markers were developed previously. All the markers linked to agronomic traits of interest in lupin were developed in the Lupin Molecular Laboratory at Department of Agriculture and Food Western Australia. Scaffold sequences from the lupin genome sequence assembly bearing these molecular markers have been deposited at Genbank (Submission number: SUB139069; Project number: PRJNA179231).

The parental line Tanjil is resistant to phomopsis stem blight (PSB) disease, while the parental line Unicrop is susceptible. The merging of PSB phenotyping data and marker genotyping data of the F_8_ population has mapped the R gene *PhtjR* for phomopsis resistance [45] in linkage group SLG11. Thirty five SNP markers were linked to the R gene within 5 cM (highlighted in blue in Table S2). Five of the SNP markers were identified as co-segregating (0 cM) with the R gene *PhtjR* (Table 3). These co-segregating markers were aligned into two scaffolds, scaffold98007 and scaffold84773 (Table S2, Table 3). Sequence analysis on these two scaffolds did not find any gene sequence homologous with known plant disease resistance genes.

Over the last 10 years, we have applied DNA fingerprinting for marker development in molecular lupin breeding, from which we have developed 16 molecular markers linked to various genes for important agronomic traits, including disease resistance genes, the early flowering gene *Ku*, the soft seed coat gene *mollis*, the pod non-shatter genes *le* and *tardus*, and the low alkaloid gene *iucundus* (Table 3). Most of these markers were insertion/deletion (indel) based DNA polymorphisms. By using the sequences of these markers to blast the draft genome assembly, we have identified one specific scaffold for each of these 16 molecular markers (Table 3). The length of the scaffolds corresponding to these 16 markers ranged from 8,191 bp to 64,039 bp (Table 3).

### Identification and Mapping of Scaffolds Containing Functional Genes

High protein seeds are unique amongst legumes and they serve as an excellent nutritional source for humans. Lupin seeds contain approximately 41% storage protein in the kernel. By using publicly available sequences of lupin storage proteins to blast our lupin genome sequence assembly, we have identified the specific scaffold for each of the storage proteins previously reported in *Lupinus angustifolius* (Table S5). An α conglutin storage protein (Genbank accession No HQ670406.1) was located on scaffold 23976, which was mapped on SLG11 and linked with two SNP markers DAFWA5526 and DAFWA6496 (Table S5). A γ conglutin storage protein (Genbank accession No HQ670417.1) was located at scaffold 84378, which was mapped on SLG2 and tagged by two SNP markers DAFWA6609 and DAFWA8013 (Table S5).

An interesting feature of lupin is the amino acid composition of storage proteins in seeds, which are low in methionine, and very high in arginine [5]. We have mapped annotated genes from both lupin and soybean genomes to methionine and arginine metabolic pathways (http://www.genome.jp/kaas-bin/kaas_main?mode=partial). Arginase (EC:3.5.3.1) was mapped in the soybean urea pathway, but not mapped in the lupin urea pathway (Figure S2). Arginase catalyses the degradation of arginine to urea. A decrease in arginase will favor arginine accumulation. Methionine is a nutritionally valuable sulphur-containing amino acid. Homocysteine S-methyltransferase (EC:2.1.1.10) and cystathionine beta-lyase (EC:4.4.1.8) were mapped in soybean cystein and methionine metabolic pathways, but not mapped in lupin cystein and methionine metabolic pathways (Figure S3). The former catalyzes the formation of methionine from homocysteine, while the later generates the methionine precursor from cystathionine. A decrease in the activities of these two enzymes could possibly reduce the production of methionine (Figure S3).

Lupin seeds have unique carbohydrate properties. They contain a negligible level of starch (1.4%), but have a high content of non-starch polysaccharide and dietary fibres, and a substantial amount of pectin [5]. A large part of the non-starch polysaccharide in lupin seeds is composed of galactose [5]. Biosynthesis of galactans is catalysed by galactosyltransferases. From blast searching of the lupin genome assembly, we found that 33 lupin scaffolds carry putative galactosyltransferases genes (Table S6). Two of the genes were mapped to SLG1 and SLG11 on scaffold 2992 and scaffold 72257, respectively. Degradation of galactans is catalysed by galactosidases and other hydroxylating enzymes. Putative α-galactosidase and β-galctosidase genes were identified (Table S6). Seven of them were mapped to SLG1, SLG4, SLG7 and SLG9 with most of the genes on SLG4 (Table S6).

## Discussion

In this study, the genome size of *L. angustifolius* was estimated at 1.153 Gb, and the sequencing data obtained from the whole genome shotgun approach represented a 26.9X coverage of the lupin genome. In plant genomes, there is often a considerable amount of sequence duplication [33], [34], [35]. The repetitive sequences in lupin were estimated at 50% in this study. When a section of DNA sequence appears more than once in a genome, it is accounted for only once in the genome sequence assembly unless the scaffold bearing the sequence is stretched beyond the duplicated region, which then enables the differentiation of the duplication. Therefore, when a genome sequence is incomplete and is at draft stage, the length of the genome sequence assembly will typically be smaller than the genome size. For example, the length of the cucumber draft genome sequence (243.5 Mbp) was approximately 66% of the genome size (367 Mbp) [46], and the length of the draft genome sequence of *Lotus japonicas* (315 Mbp) was 67% of the genome size (472 Mbp) [36]. The length of the lupin genome sequence assembly achieved in this study was approximately 52% of the lupin genome, which is a clear indication that our sequence assembly is at draft stage and is incomplete. The gene annotation results presented in this study were based on blast analysis to related annotated genomes. Due to the fragmented draft assembly, the estimated gene number in lupin was preliminary. However, this study has been the first to provide the genome sequences and the gene content of this “orphan” legume crop.

The genetic map constructed in this study possessed several major advantages over previously reported maps in *Lupinus angustifolius*. Firstly, the number of markers on the new map is several times greater than those in previously reported maps [24]–[26]. The large number of markers on the map serves to provide higher resolution land marks for the lupin genome. This will provide lupin geneticists and breeders with a broader suite of options to choose markers for a wide range of research purposes [43]. Secondly, all the 8,246 markers in our new map are DNA sequence-defined. These can easily and unambiguously be transferred and interpreted in any germplasm of *L. angustifolius*, and are useful for comparative genomic studies with other plant species. Thirdly, the DNA markers in our current map were developed from the domesticated x domesticated cross, and are more useful and relevant to the modern lupin breeding programs than the markers developed from the historical wild x domesticated cross. Fourthly, 7,563 markers in our current map are SNP markers, which are compatible with modern SNP genotyping platforms for high-throughput implementation in molecular breeding and genetics studies. However, the 8,246 sequence-defined markers were mapped only on 1,517 loci in the map (average 5.4 markers per locus); and there is thus room for improvement in our map. For example, the map still has four “gaps” (one gap each in SLG3, SLG4, SLG7 and SLG9, respectively) if we use the threshold of 20 cM without a marker [24]. One possible reason for these limitations is that the two parental cultivars of the mapping population were both Australian domesticated cultivars which share close kinship, and no polymorphism would be detected in the chromosome regions wherever both parents preserved the same DNA sequences during the breeding process. Furthermore, we were unable to map the domestication genes in our map due to the fact that the two parents were both domesticated cultivars, and the domestication genes did not segregate in the mapping population employed in this study.

The three genetic linkage maps published previously for *L. angustifolius* [24]–[26] were based on the same F_8_ RIL mapping population from the cross between a wild lupin accession (P27255) and a domesticated breeding line (83A:476). Attempts were made to clarify the relationship between the old maps and the new map by obtaining sequences of previously mapped markers and blasting them against the lupin genome assembly to identify their corresponding scaffolds. Unfortunately, the results were complex and inconclusive due to two major difficulties. Firstly, the majority of the markers in previous maps were anonymous (MFLP markers, AFLP markers and RFLP markers) without sequence information, which greatly limited their usefulness in sequence comparison. Secondly, most of the markers with sequence information in previous maps were based on genes originating from ESTs/cDNA of lupin, or from gene sequences of *Medicago truncatula* or *Lotus japonicus* [26,26], which lack specificity in sequence comparison due to gene duplications. Examples of several markers developed from one gene sequence being mapped into different linkage groups in previous maps were abundant. For example, when one cDNA sequence from lupin (Genbank accession number DT454398) was used as a probe in RFLP tests, four markers (UWA097a, UWA097b, UWA097c and UWA097d) were detected; these four markers were mapped at four loci on three linkage groups (UWA097b on linkage group NLL-06, UWA097c on NLL-13, and UWA097a and UWA097d at two loci on NLL-07) [26], suggesting that there were at least four copies of the gene of the cDNA DT454398 in the lupin genome. The available sequences in previous maps did not allow us to produce a meaningful alignment of previous maps and our sequence-defined map. At the current time, research work is under way by the authors to select 768 SNP markers (8 plates of primers each containing 96 markers) from our sequence-defined map for an even genome coverage to formulate a “Lupin SNP Array” using the Fluidigm nanoflidic array genotyping platform [47]. This array will be used to screen the wild x domesticated F_8_ mapping population used in previous maps, and the resultant dataset should allow the reconciliation of previous lupin maps with the current map in the near future.

In genetic mapping, the number of molecular markers mapped for the agronomic genes for seed coat colour, *Lanr1* and *PhtjR* within the genetic distance of 5 cM were 63, 37 and 35, respectively. Each of these three genes had 24, 2 and 5 co-segregating markers. The two R genes were mapped to different linkage groups, with the gene *Lanr1* more toward the distal, and the gene *PhtjR* more towards the proximal of the chromosomes. A candidate R gene was identified for *Lanr1* (conferring resistance to anthracnose) based on the gene structure of TIR-NBS-LRR, and correlation with disease phenotypes on the F_8_ population containing 190 RILs. However, the perfect linkage and annotation of the gene are not conclusive proof of the relationship between gene function and the expression of anthracnose disease resistance in lupin, and more research is required to confirm this. The successful identification of large scaffold sequences for thousands of RAD-sequencing derived markers and for previously developed small-sized DNA markers is testimony to the applicability of our draft assembly (obtained from a whole genome shotgun sequence dataset) in marker development for lupin breeding. In traditional marker development by DNA fingerprinting methods (such as RAPD, AFLP and MFLP), the DNA markers recovered from the gels must go through a tedious process of DNA fragment isolation, PCR amplification, cloning and sequencing to determine the DNA sequences to enable the design of sequence-specific primers [15], [48]. Sometimes marker conversion may still remain problematic even after the marker bands are sequenced, particularly for dominant markers, and for markers resulting from DNA variations from the restriction sites targeted by the restriction enzymes employed in DNA fingerprinting. In these cases, further DNA sequence extension after sequencing is required [38], [49]. With the draft genome assembly reported in this study, lupin breeders and molecular geneticists are now able to blast search for large scaffold DNA sequences from small-sized candidate markers generated from DNA fingerprinting. This will greatly facilitate the primer design and marker conversion for the development of cost-effective PCR-based markers for molecular lupin breeding.

The gene annotation results were based on blast analysis to related annotated genomes. Due to the fragmented draft assembly achieved in this study, the estimated gene number was preliminary. However, the draft assembly is still valuable in studying the functional genes in lupin. For example, previous studies on lupin storage proteins were based upon gene expression (including mRNA and cDNA translations) [50], gene product (protein) isolation [51], or homology with storage proteins from other species [52]. In this study, we identified the scaffold sequences for each of the lupin storage proteins, which, for the first time, provided the genomic DNA sequences flanking the protein genes. These sequences might be valuable for future study of the gene structure and gene regulation (such as promoters) in relation to the storage proteins in *Lupinus angustifolius.* Lupin seeds also contain a high level of galactose [5]. At the current time, little is known of the genes or enzymes controlling the biosynthesis of galactose-containing polysaccharides in plants. Lupin may serve as a good model for the study of the biosynthesis of galactose-containing polysaccharides, for which the genomic resources reported in this study become useful. Although pectin biosynthesis has been intensively studied in other plant species such as in *Arabidopsis* [53], the regulation of enriched pectin biosynthesis is unknown in lupin. We have identified over 19 galacturonosyltransferase genes in the lupin genomes (Table S6). These genes might play an important role in pectin biosynthesis. Five of them were mapped to LSG1, SLG2, SLG8 and SLG14 (Table S6). Furthermore, lupin seeds seem to use non-starch polysaccharides for energy storage, which is unlike other grain species where starch is the major form of energy storage. The high level of non-starch polysaccharides in lupin seeds is beneficial in human diets. The sequencing of the lupin genome is thus the first step towards a better understanding of the biosynthesis of these non-starch polysaccharides.

### Conclusions

Lupin (*Lupinus angustifolius* L.) is the most recently domesticated crop in major agricultural cultivation. It still remains as a young, minor crop among the world grain crops in terms of cultivation acreage. Lupin seeds are valued for their high protein and dietary fibre, as well as their low fat and starch content. Great potential exists for its broader cultivation and utilization as a food to provide important health benefits to meet the dietary needs of humans in modern life. Before this study, there was little published knowledge of the lupin genome. This is the first report of genome sequences of lupin. The draft assembly from a whole genome shotgun sequencing dataset reported in this study provides the much needed genomic resources to expedite genomic and genetic studies on this legume crop species, and will also be valuable for comparative genomic studies for other plant species. The gene annotation database (Table S1) provides, for the first time, an understanding of the gene content in lupin, which is valuable for future studies on genes, gene structure and functional genomics. Analysis of the annotated genes with metabolic pathways provided a partial understanding of some key features of lupin, such as the amino acid profile of storage proteins in seeds. The dense genetic map, including the thousands of sequence-defined SNP markers and their corresponding scaffolds (the database as presented in Table S2), provides the sign-posts for lupin genome. This will be useful for future studies of comparative genomics for other plant species, as well as for molecular genetic study and marker-assisted breeding in lupin.

Two milestone cultivars, Unicrop and Tanjil, were employed in our experiments. Unicrop was the first fully domesticated cultivar of *L angustifolius*, and is of low yield, has limited adaption to soil types, is susceptible to diseases such as phomopsis stem blight, anthracnose, grey leaf spot, and CMV. In contrast, Tanjil is high-yielding, well adapted to a wide range of soil types and climate conditions, and is resistant to all the diseases listed above. The F_8_ RILs from the cross Unicrop x Tanjil not only segregated for these agronomic traits, they may also contain novel alleles resulting from recombination breakpoints within genic sequences [54]. Once the F_8_ population has been accurately phenotyped for these traits, the molecular markers developed in this study will be able to map and pinpoint these agronomic genes of interest. The selection of cultivar Tanjil for genome sequencing in this study has ensured that all these desirable genes are present in the current draft genome assembly, which will greatly facilitate the identification, cloning and manipulation of these genes in future studies.

## Materials and Methods

### Plant Materials

Plants employed in this study were the two cultivars Tanjil and Unicrop of *Lupinus angustifolius*, and the F_8_ RIL population from the cross made between these two parental cultivars. A single plant of cultivar Tanjil was used as the pollen donor, and was crossed with a single plant of cultivar Unicrop. F_2_ seeds from a single F_1_ plant were harvested and advanced to F_8_ RILs by single seed descent with no bias. Self-pollinated seeds from the two single parental plants Unicrop and Tanjil used in the crossing were harvested separately. Plants for genome sequencing of *L. angustifolius* were from cultivar Tanjil, which were growing from the single-seed derived self-pollinated line from the above crossing. The two parental plants and the F_8_ RILs were used in SNP/indel marker discovery for genetic map construction.

### *De novo* Genome Sequencing and Annotation

Genome sequencing of *Lupinus angustifolius* was performed by the whole genome shotgun (WGS) approach [55]. Seeds of the single-seed-descent derived line of Tanjil were sown in the glasshouse. Three weeks after sowing, fully expanded leaves were harvested for DNA extraction. DNA was randomly sheared by nebulization, end-repaired with T4 DNA polymerase, and size selected by gel electrophoresis on 1% low-melting-point agarose. Two sequencing libraries of insert-size 500 bp and 800 bp were constructed according to the Illumina Inc. manufacturer instructions. The Pair-end sequencing of the sequencing libraries was performed on a HiSeq2000 platform. Genome sequence assembly was performed with the software program SOAPdenovo [31], [32] with a *K*-mer of 17. The scaffold sequences of the draft assembly from the whole genome shotgun sequencing dataset have been deposited at Genbank (Submission number “SUB139069; BioPreoject number “PRJNA179231”; website address: http://www.ncbi.nlm.nih.gov/bioproject?term=PRJNA179231).

Lupin genome annotation was performed by a homology search against the gene database of *Arabidopsis* (TAIR9, http://www.arabidopsis.org/) and *Glycine max* (Version 4.0, ftp://ftp.jgi-psf.org/pub/JGI_data/phyto-zome/v4.0/Gmax) with NCBI blast toolkit.

### Construction of a Genetic Linkage Map with Sequence-defined Markers

The two parental plants Tanjil and Unicrop, and 94 resultant F_8_ RILs were used in the genetic mapping study. The protocols of RAD sequencing were the same as Chutimanitsakun *et al.* [56], except that we used the restriction enzyme *EcoRI* (recognition site 5′-G/AATTC-3′) to replace the restriction enzyme *SbfI. EcoRI* is a more frequent cutter than *SbfI,* resulting in the detection of a larger number of markers. Ten single-end sequencing libraries (100 bp) were constructed by using the eight-nucleotide multiplex identifiers (MID) [57]. Each library contained 10 test plants. Each plant was assigned to a unique MID barcode. The RAD products from the 96 plants were processed in 10 lanes on the NGS platform HiSeq2000 (which contains 16 lanes per run). Sequencing data were segregated into each of the 96 individual plants according to their respective eight-nucleotide MID barcodes in each library [57]. The length of DNA sequences of RAD reads was 100-bp including the MID barcodes. After the RAD reads were assigned into individual plants, the eight-nucleotide MID barcode sequences were removed. The length of RAD reads used in bioinformatics analysis was 92 bp. The 92-bp RAD reads within each individual plant were clustered into read tags based on sequence similarity. Namely, RAD reads containing the same DNA sequences within each plant were placed into one read tag. Clustered tags containing more than 100 RAD reads were filtered and removed to avoid the detection of SNP markers from repetitive regions [58].

DNA sequences of RAD read tags were compared between the two parental plants. RAD reads with DNA sequences monomorphic between the two parents were filtered and removed. Only the sequence reads containing SNP markers and indel markers polymorphic between the two parents were retained. The resultant sequence reads containing SNP/indel markers were compared among the 94 RIL plants. The genotypes of the SNP/indel markers of the 94 RILs were used for the mapping study [56], [57], [59]. Several software programs were used to construct a molecular genetic linkage map with 8,246 molecular markers (Table S2), including MapManager QTX [60], MapQTL5 (www.biometris.nl) and MultiQTL (www.multiqtl.com). The original marker data were first grouped at high stringency (LOD>6), followed by distribution of small groups into large linkage groups by gradually reducing the LOD score until the linkage groups were close to the lupin chromosome numbers. The final marker order on each linkage group was verified by the software program RECORD [61].

### Integration of Assembled Scaffolds into the Genetic Linkage Map

DNA sequences of the SNP/indel markers were used to blast the draft genome assembly which resulted from the parental line Tanjil. If one unique scaffold sequence was identified from the sequence assembly which showed 100% match with the sequence of one particular SNP marker, the scaffold was aligned into the sequenced-defined lupin genetic map on the locus where the corresponding SNP marker was located.

### Identification of SNP Markers, Candidate Genes and Functional Genes of Agronomic Interest

Cultivar Tanjil contains a major gene, designated as *Lanr1*, against anthracnose disease [14], which has been extensively used in the Australia national lupin breeding program to combat the disease. The R gene *Lanr1* is polymorphic between the two parental lines Tanjil (resistant) and Unicrop (susceptible), and was segregating among the 94 F_8_ RILs employed for genetic mapping in this study. The parental lines and the 94 F_8_ RILs were phenotyped for anthracnose disease resistance based on the method described Yang *et al.* [14]. The anthracnose disease phenotyping data and the SNP/indel marker genotyping data from RAD sequencing on the 94 F_8_ RILs were combined and subjected to genetic linkage analysis by software MapManager [62] to determine the location of the R gene on the sequence-defined lupin linkage map. SNP markers co-segregating (0 cM) with the R gene phenotype were identified. DNA sequences of scaffolds bearing the co-segregating SNP markers were analyzed by the GenScan server software (http://genes.mit.edu/GENSCAN.html) [41] to search for the presence of candidate disease resistance homologs linked to disease resistance.

Similarly, Tanjil contains an R gene *PhtjR* conferring resistance against phomopsis stem blight disease [45], while Unicrop is susceptible to the disease. The parental lines and the 94 F_8_ RILs used for genetic mapping in this study were phenotyped for phomopsis stem blight disease based on the method described by Shankar *et al*. [62]. The disease phenotyping data were combined with the SNP mapping data for linkage analysis by the software program MapManager to determine the locus of the R gene in the sequence-defined map. Co-segregating SNP markers and candidate disease resistance homologs linked to disease resistance *PhtjR* were identified based on the same strategy as above.

The parental cultivar Tanjil has dark brown speckles on the seed coat, while the seed coat of parental line Unicrop is white. The resultant 94 F_8_ RILs employed in genetic mapping in this study segregated for this agronomic trait. Seed coat colour of the parental lines and the 94 RILs was visually inspected and recorded. The seed coat colour phenotyping data on the 94 RILs were combined with SNP/indel genotyping data for genetic linkage analysis to identify the markers linked to the seed coat colour gene. The DNA sequences of the SNP/indel markers co-segregating with the seed coat colour gene were used to blast the lupin genome sequence assembly to identify scaffolds linked with the seed coat colour gene.

In the previous 10 years, we have developed 16 molecular markers linked to various agronomic genes of interest in marker development by DNA finger printing (Table 3). The DNA sequences of these 16 markers were used to blast the lupin draft assembly from the whole genome shotgun sequencing dataset to search for the corresponding scaffolds containing DNA sequences of these markers.

The sequences of lupin storage proteins publicly available were used to fetch nucleotide sequences from Genbank (http://www.ncbi.nlm.nih.gov/genbank/). These nucleotide sequences were used as queries to blast lupin genome sequence databases to identify scaffolds for each lupin storage protein by a standalone blastn.exe program (ftp://ftp.ncbi.nlm.nih.gov/blast/executables/blast/LATEST/). Lupin genome sequences (scaffolds) were mapped to KEGG metabolic pathways (http://www.genome.jp/kaas-bin/kaas_main) by using the KEGG automatic annotation server to explore the lupin genes controlling the low level of methionine and the high level of arginine in storage proteins in the seeds [5]. For identification of carbohydrate metabolic genes, the lupin gene annotation dataset (Table S1) was used to match the genes in soybean Gmax-109-annotation table (ftp://ftp.jgi-psf.org/pub/compgen/phytozome/v8.0/Gmax_v1.0/). The carbohydrate metabolic genes were identified by searching the matches with key words including glucose-, galactose-, xylose- arabinose-containing polymers, pectin, cellulose, and glycosyltransferase.

## Supporting Information

Figure S1 **The candidate R gene linked to anthracnose disease resistance in** ***Lupinus angustifolius*** **.**
(TIF)

Figure S2 **Comparison of arginine metabolic pathways in soybean and lupin.** Arginase (EC:3.5.3.1) was mapped in the soybean urea pathway, but not mapped in the lupin urea pathway.
(TIF)

Figure S3 **Comparison of methionine metabolic pathways in soybean and lupin.** Homocysteine S-methyltransferase and cystathionine beta-lyase were mapped at the cystein and methionine metabolic pathways in soybean, but not mapped at the cystein and methionine metabolic pathways in lupin.
(TIF)

Table S1 **Gene annotation of the genome sequence assembly of** ***Lupinus angustifolius*** **.** The 57,807 annotated genes and their positions in the corresponding scaffolds are listed.
(XLSX)

Table S2 **Genetic linkage map of** ***Lupinus angustifolius*** **constructed based on 8,246 sequence-defined molecular markers.** SNP markers are named with a prefix of “DAFWA”; and indel markers are named with a prefix of “iDAFWA”. The first nucleotides of the SNPs in parentheses in the RAD reads were from Unicrop, the second nucleotides in parentheses were from Tanjil. The numbers following ":" after scaffold names indicate the nucleotide positions of the SNP markers in the scaffold sequences. The insertion/deletion nucleotides of indel markers are noted after the scaffold names.
(XLSX)

Table S3 **List of the annotated genes on the 4,214 scaffolds integrated on the sequence-defined linkage map of** ***Lupinus angustifolius.***
(XLSX)

Table S4 **Validation of SNP marker DAFWA213 linked to the R gene** ***Lanr1*** **conferring resistance to anthracnose disease using Fluidigm SNP genotyping platform.** R = presence of the R gene *Lanr1*, S = absence of R gene *Lanr1*. Presence or absence of the R gene *Lanr1* on cultivars is adapted from You *et al*. [38]. Genotype A:A = homozygous genotype of the marker DAFWA213 linked to the disease resistance allele; G:G = homozygous genotype of the marker DAFWA213 linked to the disease susceptible allele.
(XLSX)

Table S5 **Identification of scaffolds containing seed storage protein genes in** ***Lupinus angustifolius.*** Storage protein genes showing SNP markers indicating that the scaffold bearing the gene sequences were integrated into the sequence-defined genetic map.
(DOCX)

Table S6 **Blast search of the genome sequence assembly for carbohydrate metabolic genes in** ***Lupinus angustifolius.*** Genes showing SNP markers indicating that the scaffolds bearing the gene sequence were integrated into the sequenced-defined map.
(DOCX)
