# Supplementary figures and images for: Draft Genome Sequence, and a Sequence-Defined Genetic Linkage Map of the Legume Crop Species Lupinus angustifolius L
Source: PLoS One. 2013 May 29;8(5):e64799. doi: 10.1371/journal.pone.0064799 (PMC3667174; doi:10.1371/journal.pone.0064799)

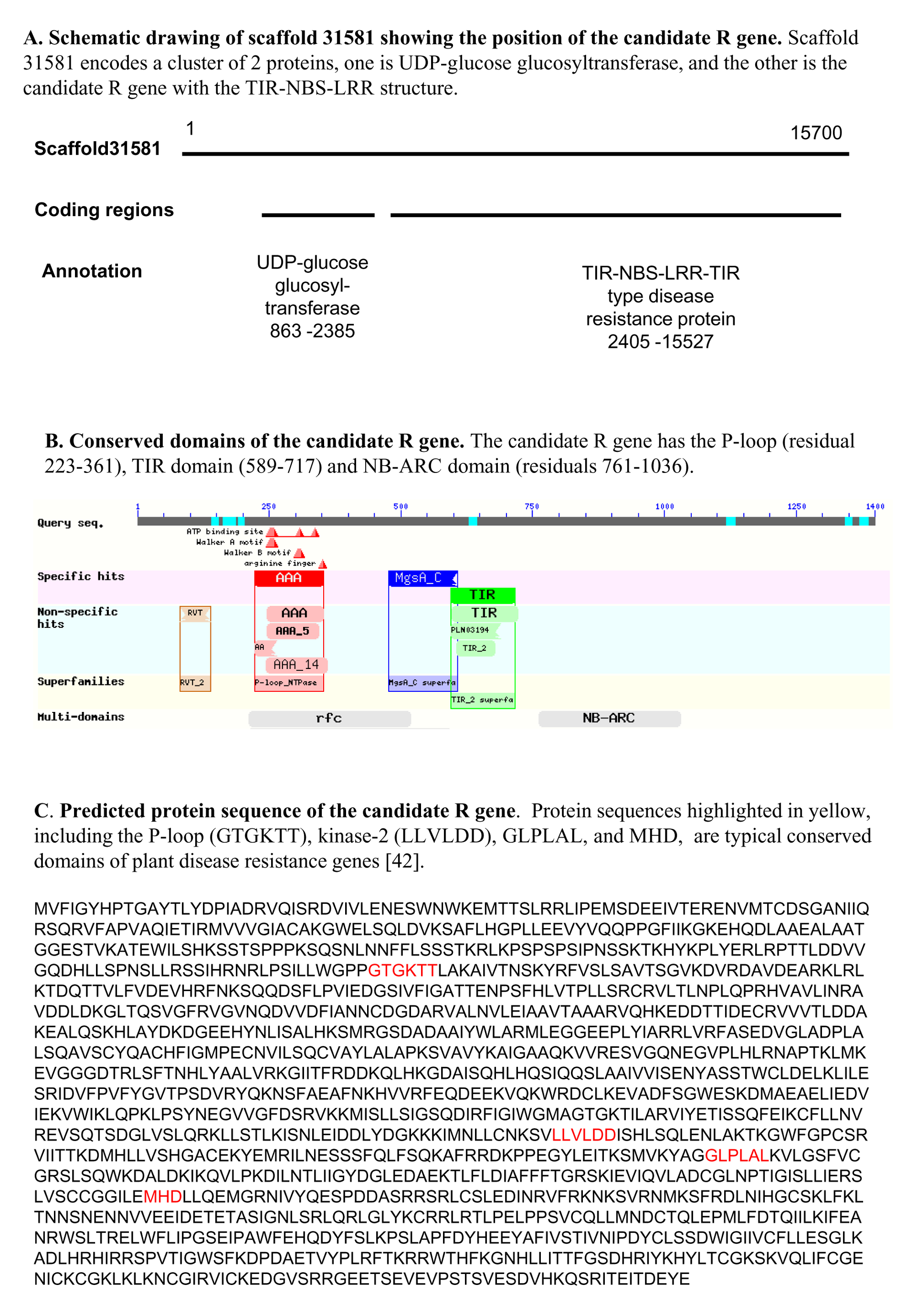

Supplement: Figure S1 — The candidate R gene linked to anthracnose disease resistance in Lupinus angustifolius . (TIF) [file pone.0064799.s001.tif]

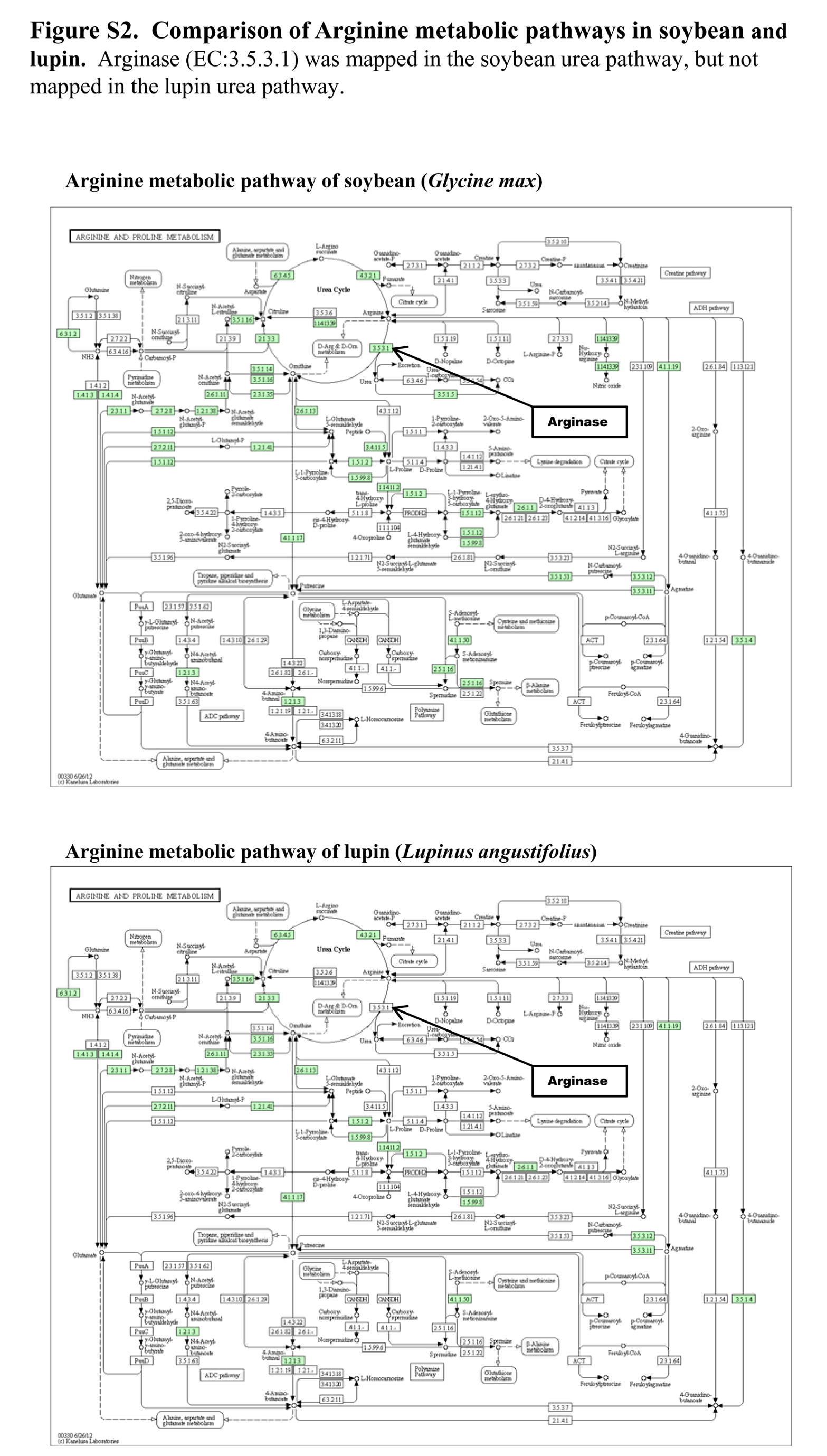

Supplement: Figure S2 — Comparison of arginine metabolic pathways in soybean and lupin. Arginase (EC:3.5.3.1) was mapped in the soybean urea pathway, but not mapped in the lupin urea pathway. (TIF) [file pone.0064799.s002.tif]

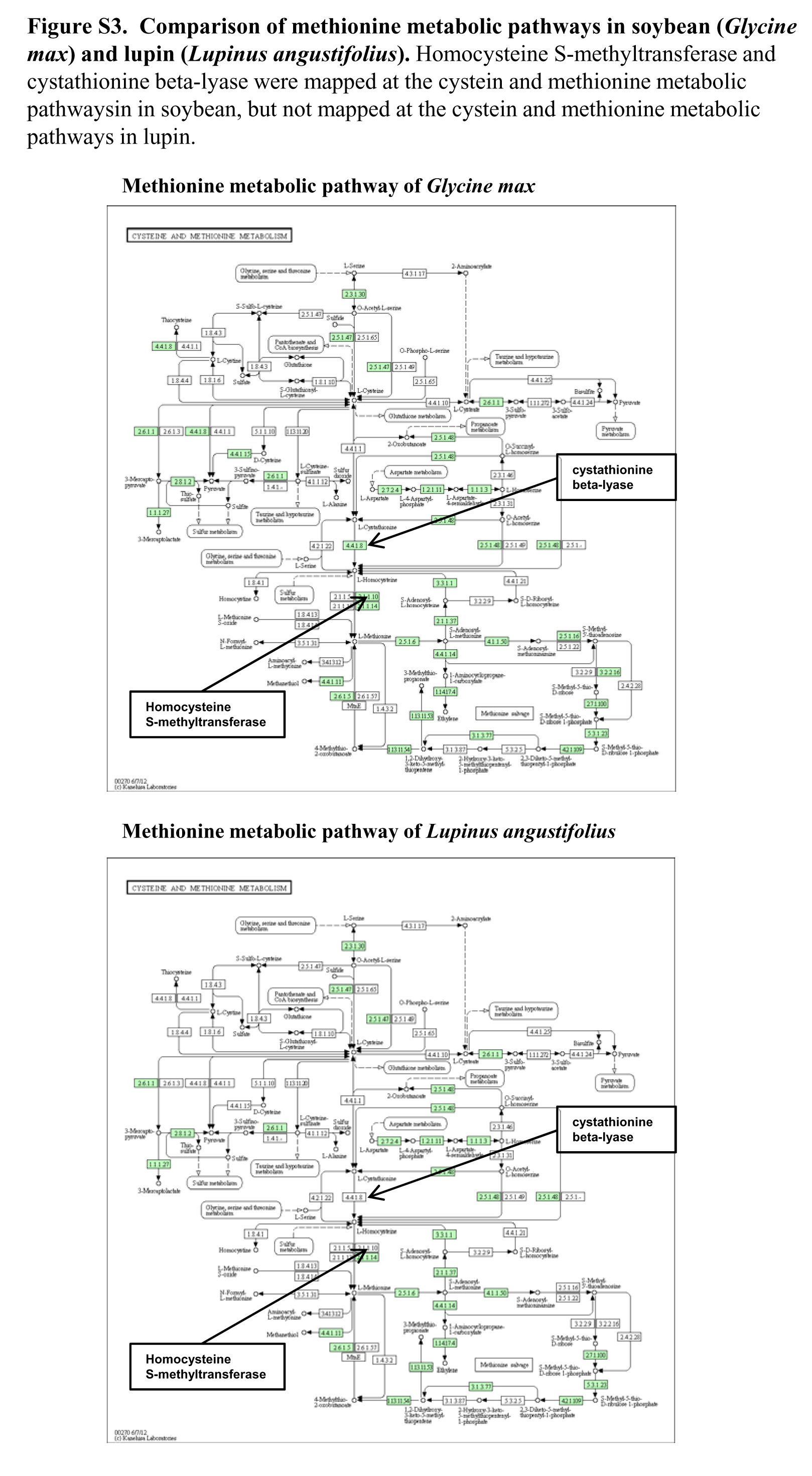

Supplement: Figure S3 — Comparison of methionine metabolic pathways in soybean and lupin. Homocysteine S-methyltransferase and cystathionine beta-lyase were mapped at the cystein and methionine metabolic pathways in soybean, but not mapped at the cystein and methionine metabolic pathways in lupin. (TIF) [file pone.0064799.s003.tif]
