# Supplementary material for: Draft Genome Sequence, and a Sequence-Defined Genetic Linkage Map of the Legume Crop Species Lupinus angustifolius L
Source: PLoS One. 2013 May 29;8(5):e64799. doi: 10.1371/journal.pone.0064799 (PMC3667174; doi:10.1371/journal.pone.0064799)
Supplement: Table S5 — Identification of scaffolds containing seed storage protein genes in Lupinus angustifolius. Storage protein genes showing SNP markers indicating that the scaffold bearing the gene sequences were integrated into the sequence-defined genetic map. (DOCX) [file pone.0064799.s008.docx]

**Table S5 Identification of scaffolds containing seed storage protein genes in *Lupinus angustifolius.*** Storage protein genes showing SLG and SNP markers indicate that the scaffolds bearing the gene sequences were integrated into the sequence-defined genetic map.

| Conglutin types | Genbank accession | Scaffold  Name | Scaffold  Size (bp) | SLG | SNP marker  Name |
| --- | --- | --- | --- | --- | --- |
| Alpha 1 | HQ670406.1 | scaffold23976 | 37,445 | SLG11 | DAFWA5526  DAFWA6496 |
| Alpha 2 | HQ670407.1 | scaffold20322 | 39,446 |  |  |
| Alpha 3 | HQ670408.1 | scaffold93856 | 16,601 |  |  |
| Alpha 3 | FJ263366.1 | scaffold93856 | 16,601 |  |  |
| Beta 1 | HQ670409.1 | scaffold75874 | 6,741 |  |  |
| Beta 1 | EF455725.1 | scaffold75874 | 6,741 |  |  |
| Beta 2 | HQ670410.1 | scaffold48545 | 7,148 |  |  |
| Beta 3 | HQ670411.1 | scaffold40379 | 5,007 |  |  |
| Beta 4 | HQ670412.1 | scaffold65390 | 20,319 |  |  |
| Beta 5 | HQ670413.1 | scaffold98484 | 3,596 |  |  |
| Beta 6 | HQ670414.1 | scaffold65390 | 20,319 |  |  |
| Beta 7 | HQ670415.1 | scaffold65390 | 20,319 |  |  |
| Beta 7 | EF455724.1 | scaffold65390 | 20,319 |  |  |
| Gamma 1 | HQ670416.1 | scaffold54922 | 8,209 |  |  |
| Gamma 1 | L39786.1 | scaffold54922 | 8,209 |  |  |
| Gamma 2 | HQ670417.1 | scaffold84378 | 34,665 | SLG2 | DAFWA8013 |
| Delta 1 | HQ670418.1 | C28537352 | 871 |  |  |
| Delta 2 | HQ670419.1 | scaffold53836 | 4,036 |  |  |
| Delta2 | X53523.1 | scaffold53836 | 4,036 |  |  |
| Delta 3 | HQ670420.1 | C28042539 | 234 |  |  |
| Delta 4 | HQ670421.1 | scaffold3418 | 39,716 |  |  |
