# Supplementary material for: Draft Genome Sequence, and a Sequence-Defined Genetic Linkage Map of the Legume Crop Species Lupinus angustifolius L
Source: PLoS One. 2013 May 29;8(5):e64799. doi: 10.1371/journal.pone.0064799 (PMC3667174; doi:10.1371/journal.pone.0064799)
Supplement: Table S6 — Blast search of the genome sequence assembly for carbohydrate metabolic genes in Lupinus angustifolius. Genes showing SNP markers indicating that the scaffolds bearing the gene sequence were integrated into the sequenced-defined map. (DOCX) [file pone.0064799.s009.docx]

**Table S6 Blast search of the genome sequence assembly for carbohydrate metabolic genes in *Lupinus angustifolius.*** Genes showing SLG and SNP markers indicate that the scaffolds bearing the gene sequence were integrated into the sequenced-defined map.

| **Scalffold name** | **Annotation** | | | | **SLG** | | | **Marker names** | | |
| --- | --- | --- | --- | --- | --- | --- | --- | --- | --- | --- |
| **Galactan biosynthesis** | | | | |  | | |  | | |
| scaffold71643 | | Galactosyl transferase GMA12/MNN10 family protein | | | | | | | |  |
| scaffold66319 | | Galactosyl transferase GMA12/MNN10 family protein | | | | | | | |  |
| scaffold84627 | | Galactosyl transferase GMA12/MNN10 family protein | | | | | | | |  |
| scaffold94955 | | Galactosyl transferase GMA12/MNN10 family protein | | | | | | | |  |
| scaffold1671 | | Galactosyltransferase family protein | |  | | |  | | |  |
| scaffold92549 | | Galactosyltransferase family protein | |  | | |  | | |  |
| scaffold30224 | | Galactosyltransferase family protein | |  | | |  | | |  |
| scaffold2992 | | Galactosyltransferase family protein | | SLG1 | | | AntjM2 | | |  |
| scaffold2992 | | Galactosyltransferase family protein | | SLG1 | | | DAFWA6620 | | |  |
| scaffold72257 | | Galactosyltransferase family protein | | SLG11 | | | DAFWA6249 | | |  |
| scaffold93179 | | Galactosyltransferase family protein | |  | | |  | | |  |
| scaffold42595 | | Galactosyltransferase family protein | |  | | |  | | |  |
| scaffold428 | | Galactosyltransferase family protein | |  | | |  | | |  |
| scaffold38882 | | Galactosyltransferase family protein | |  | | |  | | |  |
| scaffold7907 | | Galactosyltransferase family protein | |  | | |  | | |  |
| scaffold97670 | | Galactosyltransferase family protein | |  | | |  | | |  |
| scaffold22891 | | Galactosyltransferase family protein | |  | | |  | | |  |
| scaffold19344 | | Galactosyltransferase family protein | |  | | |  | | |  |
| C28716854 | | Galactosyltransferase family protein | |  | | |  | | |  |
| scaffold2965 | | Galactosyltransferase family protein | |  | | |  | | |  |
| scaffold3770 | | Galactosyltransferase family protein | |  | | |  | | |  |
| scaffold20622 | | Galactosyltransferase family protein | |  | | |  | | |  |
| scaffold96325 | | Galactosyltransferase family protein | |  | | |  | | |  |
| scaffold57641 | | Galactosyltransferase family protein | |  | | |  | | |  |
| scaffold33715 | | Galactosyltransferase family protein | |  | | |  | | |  |
| scaffold77013 | | Galactosyltransferase family protein | |  | | |  | | |  |
| scaffold48513 | | Galactosyltransferase family protein | |  | | |  | | |  |
| scaffold47824 | | Galactosyltransferase family protein | |  | | |  | | |  |
| scaffold92081 | | Galactosyltransferase family protein | |  | | |  | | |  |
| scaffold11466 | | Galactosyltransferase family protein | |  | | |  | | |  |
| scaffold71952 | | Galactosyltransferase family protein | |  | | |  | | |  |
| scaffold97243 | | Galactosyltransferase family protein | |  | | |  | | |  |
| scaffold7791 | | Galactosyltransferase family protein | |  | | |  | | |  |
| scaffold11638 | | Galactosyltransferase family protein | |  | | |  | | |  |
| **Galactan hydrolysis** | | | |  | | |  | | |  |
| scaffold89129 | | alpha-galactosidase 2 | |  | | |  | | |  |
| scaffold2022 | | alpha-galactosidase 2 | |  | | |  | | |  |
| scaffold26984 | | alpha-galactosidase 2 | |  | | |  | | |  |
| scaffold28589 | | alpha-galactosidase 2 | |  | | |  | | |  |
| scaffold21535 | | beta-galactosidase 10 | |  | | |  | | |  |
| scaffold25426 | | beta-galactosidase 12 | |  | | |  | | |  |
| scaffold48561 | | beta-galactosidase 17 | | SLG4 | | | iDAFWA656 | | |  |
| scaffold48561 | | beta-galactosidase 17 | | SLG4 | | | DAFWA6654 | | |  |
| scaffold10163 | | beta-galactosidase 3 | |  | | |  | | |  |
| scaffold34199 | | beta-galactosidase 3 | |  | | |  | | |  |
| scaffold54725 | | beta-galactosidase 3 | |  | | |  | | |  |
| scaffold95598 | | beta-galactosidase 3 | | SLG9 | | | DAFWA8129 | | |  |
| scaffold95598 | | beta-galactosidase 3 | | SLG9 | | | DAFWA1401 | | |  |
| scaffold936 | | beta-galactosidase 3 | |  | | |  | | |  |
| scaffold47980 | | beta-galactosidase 3 | |  | | |  | | |  |
| scaffold47142 | | beta-galactosidase 3 | |  | | |  | | |  |
| scaffold13641 | | beta-galactosidase 5 | |  | | |  | | |  |
| scaffold21927 | | beta-galactosidase 5 | |  | | |  | | |  |
| scaffold71273 | | beta-galactosidase 7 | |  | | |  | | |  |
| scaffold91038 | | beta-galactosidase 7 | | SLG1 | | | DAFWA7233 | | |  |
| scaffold77921 | | beta-galactosidase 8 | | SLG4 | | | DAFWA1958 | | |  |
| scaffold77921 | | beta-galactosidase 8 | | SLG4 | | | DAFWA683 | | |  |
| scaffold77921 | | beta-galactosidase 8 | | SLG4 | | | DAFWA1572 | | |  |
| scaffold67899 | | beta-galactosidase 8 | |  | | |  | | |  |
| scaffold89508 | | beta-galactosidase 8 | | SLG7 | | | DAFWA6648 | | |  |
| scaffold89508 | | beta-galactosidase 8 | | SLG7 | | | DAFWA7405 | | |  |
| scaffold89508 | | beta-galactosidase 8 | | SLG7 | | | DAFWA2067 | | |  |
| scaffold49286 | | beta galactosidase 1 | | SLG4 | | | DAFWA8402 | | |  |
| scaffold61537 | | beta galactosidase 1 | |  | | |  | | |  |
| scaffold95935 | | beta galactosidase 9 | |  | | |  | | |  |
| C28703208 | | beta galactosidase 9 | |  | | |  | | |  |
| scaffold24548 | | beta galactosidase 9 | |  | | |  | | |  |
| **Cellulose Biosynthesis** | | | |  | | |  | | |  |
| C28753894 | | cellulose synthase 1 | |  | | |  | | |  |
| scaffold98153 | | cellulose synthase 1 | |  | | |  | | |  |
| scaffold26856 | | cellulose synthase 6 | |  | | |  | | |  |
| scaffold87609 | | cellulose synthase 6 | |  | | |  | | |  |
| scaffold77336 | | cellulose synthase 6 | |  | | |  | | |  |
| scaffold94513 | | cellulose synthase 6 | |  | | |  | | |  |
| scaffold36074 | | cellulose synthase 6 | |  | | |  | | |  |
| scaffold64353 | | cellulose synthase 6 | |  | | |  | | |  |
| scaffold88213 | | cellulose synthase A4 | | SLG3 | | | DAFWA1860 | | |  |
| scaffold88213 | | cellulose synthase A4 | | SLG3 | | | DAFWA3353 | | |  |
| scaffold88213 | | cellulose synthase A4 | | SLG3 | | | DAFWA1730 | | |  |
| scaffold17741 | | cellulose synthase A4 | |  | | |  | | |  |
| scaffold85715 | | Cellulose synthase family protein | | SLG4 | | | DAFWA3848 | | |  |
| scaffold18216 | | Cellulose synthase family protein | |  | | |  | | |  |
| scaffold63375 | | cellulose synthase family protein | |  | | |  | | |  |
| scaffold68825 | | Cellulose synthase family protein | |  | | |  | | |  |
| scaffold70626 | | Cellulose synthase family protein | |  | | |  | | |  |
| scaffold70196 | | Cellulose synthase family protein | |  | | |  | | |  |
| scaffold82169 | | Cellulose synthase family protein | |  | | |  | | |  |
| scaffold23824 | | Cellulose synthase family protein | |  | | |  | | |  |
| **HemiCellulose biosynthesis** | | |  | | |  | | |  |  |
| scaffold3328 | | cellulose synthase like D4 | |  | | |  | | |  |
| scaffold39387 | | cellulose synthase like D4 | | SLG20 | | | DAFWA7520 | | |  |
| scaffold8374 | | cellulose synthase like E1 | |  | | |  | | |  |
| scaffold28041 | | cellulose synthase like E1 | |  | | |  | | |  |
| scaffold1820 | | cellulose synthase like G1 | |  | | |  | | |  |
| C28634930 | | cellulose synthase like G2 | |  | | |  | | |  |
| scaffold84882 | | cellulose synthase-like A02 | | SLG7 | | | DAFWA1377 | | |  |
| scaffold65124 | | cellulose synthase-like A02 | |  | | |  | | |  |
| scaffold84882 | | cellulose synthase-like A02 | | SLG7 | | | DAFWA6661 | | |  |
| scaffold70522 | | cellulose synthase-like A02 | |  | | |  | | |  |
| scaffold84882 | | cellulose synthase-like A02 | | SLG7 | | | iDAFWA534 | | |  |
| scaffold35157 | | cellulose synthase-like A02 | | SLG1 | | | DAFWA7012 | | |  |
| scaffold35157 | | cellulose synthase-like A02 | | SLG1 | | | DAFWA6646 | | |  |
| scaffold35157 | | cellulose synthase-like A02 | | SLG1 | | | DAFWA8678 | | |  |
| scaffold84882 | | cellulose synthase-like A02 | | SLG7 | | | DAFWA6684 | | |  |
| scaffold84882 | | cellulose synthase-like A02 | | SLG7 | | | DAFWA4082 | | |  |
| scaffold90138 | | cellulose synthase-like B4 | |  | | |  | | |  |
| scaffold60932 | | cellulose synthase-like B4 | |  | | |  | | |  |
| scaffold75580 | | cellulose synthase-like B4 | |  | | |  | | |  |
| scaffold75686 | | cellulose synthase-like B4 | |  | | |  | | |  |
| scaffold97687 | | cellulose synthase-like D1 | |  | | |  | | |  |
| scaffold73927 | | cellulose synthase-like D3 | |  | | |  | | |  |
| scaffold47935 | | cellulose synthase-like D3 | |  | | |  | | |  |
| scaffold45357 | | cellulose synthase-like D3 | |  | | |  | | |  |
| scaffold84654 | | Cellulose-synthase-like C12 | |  | | |  | | |  |
| scaffold88679 | | Cellulose-synthase-like C12 | |  | | |  | | |  |
| scaffold91140 | | Cellulose-synthase-like C12 | |  | | |  | | |  |
| scaffold70917 | | Cellulose-synthase-like C12 | |  | | |  | | |  |
| scaffold52979 | | Cellulose-synthase-like C12 | |  | | |  | | |  |
| scaffold79191 | | Cellulose-synthase-like C12 | |  | | |  | | |  |
| scaffold97660 | | Cellulose-synthase-like C12 | |  | | |  | | |  |
| scaffold71133 | | Cellulose-synthase-like C12 | |  | | |  | | |  |
| scaffold20757 | | Cellulose-synthase-like C4 | |  | | |  | | |  |
| scaffold48089 | | Cellulose-synthase-like C5 | |  | | |  | | |  |
| scaffold42844 | | Cellulose-synthase-like C6 | |  | | |  | | |  |
| scaffold83766 | | Cellulose-synthase-like C6 | |  | | |  | | |  |
| **Pectin biosynthesis and degradation** | | | |  | | |  | | |  |
| scaffold77431 | | Pectin lyase-like superfamily protein | | SLG4 | | | DAFWA8236 | | |  |
| scaffold69954 | | Pectin lyase-like superfamily protein | |  | | |  | | |  |
| scaffold77431 | | Pectin lyase-like superfamily protein | | SLG4 | | | DAFWA6152 | | |  |
| C28542412 | | Pectin lyase-like superfamily protein | |  | | |  | | |  |
| scaffold85000 | | Pectin lyase-like superfamily protein | |  | | |  | | |  |
| scaffold4901 | | Pectin lyase-like superfamily protein | | SLG2 | | | DAFWA4571 | | |  |
| scaffold80858 | | Pectin lyase-like superfamily protein | |  | | |  | | |  |
| scaffold96299 | | Pectin lyase-like superfamily protein | |  | | |  | | |  |
| C27517231 | | Pectin lyase-like superfamily protein | |  | | |  | | |  |
| C28504236 | | Pectin lyase-like superfamily protein | |  | | |  | | |  |
| C27523027 | | Pectin lyase-like superfamily protein | |  | | |  | | |  |
| scaffold35813 | | Pectin lyase-like superfamily protein | |  | | |  | | |  |
| scaffold60006 | | Pectin lyase-like superfamily protein | |  | | |  | | |  |
| scaffold97324 | | Pectin lyase-like superfamily protein | | SLG13 | | | DAFWA5798 | | |  |
| scaffold25145 | | Pectin lyase-like superfamily protein | |  | | |  | | |  |
| scaffold96760 | | Pectin lyase-like superfamily protein | | SLG7 | | | DAFWA5638 | | |  |
| scaffold86613 | | Pectin lyase-like superfamily protein | |  | | |  | | |  |
| scaffold33236 | | Pectin lyase-like superfamily protein | | SLG8 | | | DAFWA329 | | |  |
| scaffold90800 | | Pectin lyase-like superfamily protein | |  | | |  | | |  |
| scaffold33555 | | Pectin lyase-like superfamily protein | | SLG2 | | | DAFWA5230 | | |  |
| scaffold76840 | | Pectin lyase-like superfamily protein | |  | | |  | | |  |
| scaffold83776 | | Pectin lyase-like superfamily protein | |  | | |  | | |  |
| scaffold96760 | | Pectin lyase-like superfamily protein | | SLG7 | | | DAFWA2541 | | |  |
| scaffold10776 | | Pectin lyase-like superfamily protein | |  | | |  | | |  |
| scaffold96627 | | Pectin lyase-like superfamily protein | |  | | |  | | |  |
| scaffold34311 | | Pectin lyase-like superfamily protein | |  | | |  | | |  |
| scaffold3456 | | Pectin lyase-like superfamily protein | |  | | |  | | |  |
| scaffold31808 | | Pectin lyase-like superfamily protein | |  | | |  | | |  |
| scaffold76629 | | Pectin lyase-like superfamily protein | |  | | |  | | |  |
| scaffold83717 | | Pectin lyase-like superfamily protein | |  | | |  | | |  |
| scaffold85194 | | Pectin lyase-like superfamily protein | |  | | |  | | |  |
| scaffold98305 | | Pectin lyase-like superfamily protein | |  | | |  | | |  |
| scaffold86807 | | Pectin lyase-like superfamily protein | |  | | |  | | |  |
| scaffold80069 | | Pectin lyase-like superfamily protein | | SLG7 | | | DAFWA6507 | | |  |
| scaffold2680 | | Pectin lyase-like superfamily protein | | SLG1 | | | DAFWA3385 | | |  |
| scaffold2680 | | Pectin lyase-like superfamily protein | | SLG1 | | | DAFWA5893 | | |  |
| scaffold2680 | | Pectin lyase-like superfamily protein | | SLG1 | | | DAFWA5196 | | |  |
| scaffold80069 | | Pectin lyase-like superfamily protein | | SLG7 | | | DAFWA4688 | | |  |
| scaffold65717 | | Pectin lyase-like superfamily protein | |  | | |  | | |  |
| scaffold80069 | | Pectin lyase-like superfamily protein | | SLG7 | | | DAFWA1082 | | |  |
| scaffold53502 | | Pectin lyase-like superfamily protein | |  | | |  | | |  |
| scaffold80069 | | Pectin lyase-like superfamily protein | | SLG7 | | | DAFWA6038 | | |  |
| C28702346 | | Pectin lyase-like superfamily protein | |  | | |  | | |  |
| scaffold98347 | | Pectin lyase-like superfamily protein | |  | | |  | | |  |
| scaffold98350 | | Pectin lyase-like superfamily protein | | SLG6 | | | DAFWA1225 | | |  |
| scaffold532 | | Pectin lyase-like superfamily protein | | SLG2 | | | iDAFWA381 | | |  |
| C27863913 | | Pectin lyase-like superfamily protein | |  | | |  | | |  |
| scaffold532 | | Pectin lyase-like superfamily protein | | SLG2 | | | DAFWA4751 | | |  |
| scaffold92983 | | Pectin lyase-like superfamily protein | |  | | |  | | |  |
| C28205085 | | Pectin lyase-like superfamily protein | |  | | |  | | |  |
| C28204033 | | Pectin lyase-like superfamily protein | |  | | |  | | |  |
| scaffold65514 | | Pectin lyase-like superfamily protein | |  | | |  | | |  |
| scaffold90834 | | Pectin lyase-like superfamily protein | |  | | |  | | |  |
| scaffold92314 | | Pectin lyase-like superfamily protein | |  | | |  | | |  |
| scaffold49638 | | Pectin lyase-like superfamily protein | | SLG14 | | | DAFWA7450 | | |  |
| scaffold68746 | | Pectin lyase-like superfamily protein | | SLG17 | | | DAFWA8547 | | |  |
| scaffold25389 | | Pectin lyase-like superfamily protein | |  | | |  | | |  |
| scaffold93599 | | Pectin lyase-like superfamily protein | |  | | |  | | |  |
| scaffold64060 | | Pectin lyase-like superfamily protein | |  | | |  | | |  |
| C28406540 | | Pectin lyase-like superfamily protein | |  | | |  | | |  |
| scaffold96760 | | Pectin lyase-like superfamily protein | | SLG7 | | | DAFWA3978 | | |  |
| scaffold52451 | | Pectin lyase-like superfamily protein | |  | | |  | | |  |
| scaffold84186 | | Pectin lyase-like superfamily protein | |  | | |  | | |  |
| scaffold51663 | | Pectin lyase-like superfamily protein | |  | | |  | | |  |
| scaffold97630 | | Pectin lyase-like superfamily protein | |  | | |  | | |  |
| scaffold97638 | | Pectin lyase-like superfamily protein | |  | | |  | | |  |
| scaffold87678 | | Pectin lyase-like superfamily protein | | SLG2 | | | DAFWA7284 | | |  |
| scaffold67758 | | Pectin lyase-like superfamily protein | |  | | |  | | |  |
| scaffold84222 | | Pectin lyase-like superfamily protein | |  | | |  | | |  |
| scaffold92399 | | Pectin lyase-like superfamily protein | |  | | |  | | |  |
| C28369954 | | Pectin lyase-like superfamily protein | |  | | |  | | |  |
| scaffold5972 | | Pectin lyase-like superfamily protein | |  | | |  | | |  |
| scaffold69181 | | Pectin lyase-like superfamily protein | |  | | |  | | |  |
| scaffold79013 | | Pectin lyase-like superfamily protein | |  | | |  | | |  |
| scaffold71143 | | Pectin lyase-like superfamily protein | |  | | |  | | |  |
| scaffold74560 | | Pectin lyase-like superfamily protein | |  | | |  | | |  |
| scaffold74609 | | Pectin lyase-like superfamily protein | |  | | |  | | |  |
| scaffold89409 | | Pectin lyase-like superfamily protein | |  | | |  | | |  |
| scaffold33555 | | Pectin lyase-like superfamily protein | | SLG2 | | | DAFWA6604 | | |  |
| scaffold1240 | | Pectin lyase-like superfamily protein | |  | | |  | | |  |
| scaffold11693 | | Pectin lyase-like superfamily protein | | SLG2 | | | DAFWA6489 | | |  |
| scaffold898 | | Pectin lyase-like superfamily protein | |  | | |  | | |  |
| scaffold62426 | | Pectin lyase-like superfamily protein | | SLG6 | | | DAFWA6058 | | |  |
| scaffold44386 | | Pectin lyase-like superfamily protein | |  | | |  | | |  |
| scaffold62101 | | Pectin lyase-like superfamily protein | |  | | |  | | |  |
| scaffold38142 | | Pectin lyase-like superfamily protein | |  | | |  | | |  |
| scaffold85569 | | Pectin lyase-like superfamily protein | |  | | |  | | |  |
| scaffold92000 | | Pectin lyase-like superfamily protein | |  | | |  | | |  |
| scaffold38012 | | Pectin lyase-like superfamily protein | |  | | |  | | |  |
| scaffold61840 | | Pectin lyase-like superfamily protein | |  | | |  | | |  |
| scaffold45519 | | Pectin lyase-like superfamily protein | |  | | |  | | |  |
| scaffold92748 | | Pectin lyase-like superfamily protein | |  | | |  | | |  |
| scaffold40060 | | Pectin lyase-like superfamily protein | |  | | |  | | |  |
| scaffold88721 | | Pectin lyase-like superfamily protein | |  | | |  | | |  |
| C27865679 | | Pectin lyase-like superfamily protein | |  | | |  | | |  |
| scaffold73665 | | Pectin lyase-like superfamily protein | |  | | |  | | |  |
| scaffold41246 | | Pectin lyase-like superfamily protein | | SLG18 | | | iDAFWA243 | | |  |
| scaffold42812 | | Pectin lyase-like superfamily protein | |  | | |  | | |  |
| scaffold1712 | | Pectin lyase-like superfamily protein | |  | | |  | | |  |
| scaffold55746 | | Pectin lyase-like superfamily protein | |  | | |  | | |  |
| C28678236 | | Pectin lyase-like superfamily protein | |  | | |  | | |  |
| scaffold43558 | | Pectin lyase-like superfamily protein | |  | | |  | | |  |
| scaffold9909 | | Pectin lyase-like superfamily protein | |  | | |  | | |  |
| scaffold88836 | | Pectin lyase-like superfamily protein | |  | | |  | | |  |
| scaffold99400 | | Pectin lyase-like superfamily protein | |  | | |  | | |  |
| scaffold44126 | | Pectin lyase-like superfamily protein | |  | | |  | | |  |
| scaffold5598 | | Pectin lyase-like superfamily protein | | SLG13 | | | iDAFWA102 | | |  |
| scaffold55998 | | Pectin lyase-like superfamily protein | |  | | |  | | |  |
| scaffold94923 | | Pectin lyase-like superfamily protein | |  | | |  | | |  |
| scaffold11693 | | Pectin lyase-like superfamily protein | | SLG2 | | | DAFWA1770 | | |  |
| scaffold72094 | | Pectin lyase-like superfamily protein | |  | | |  | | |  |
| scaffold95421 | | Pectin lyase-like superfamily protein | |  | | |  | | |  |
| C28650762 | | Pectin lyase-like superfamily protein | |  | | |  | | |  |
| scaffold82738 | | Pectin lyase-like superfamily protein | |  | | |  | | |  |
| scaffold74940 | | pectin methylesterase 1 | |  | | |  | | |  |
| scaffold77251 | | pectin methylesterase 3 | |  | | |  | | |  |
| scaffold15666 | | pectin methylesterase 3 | | SLG2 | | | DAFWA4210 | | |  |
| scaffold72583 | | pectin methylesterase 3 | |  | | |  | | |  |
| scaffold62021 | | pectin methylesterase 3 | |  | | |  | | |  |
| scaffold3348 | | pectin methylesterase inhibitor 1 | |  | | |  | | |  |
| scaffold27292 | | pectin methylesterase inhibitor 1 | |  | | |  | | |  |
| scaffold20829 | | pectin methylesterase inhibitor 1 | |  | | |  | | |  |
| scaffold34511 | | pectin methylesterase inhibitor 1 | |  | | |  | | |  |
| scaffold67851 | | pectin methylesterase inhibitor 1 | |  | | |  | | |  |
| scaffold28908 | | pectin methylesterase inhibitor 1 | |  | | |  | | |  |
| scaffold98430 | | pectin methylesterase inhibitor 1 | |  | | |  | | |  |
| scaffold98417 | | pectin methylesterase inhibitor 1 | |  | | |  | | |  |
| scaffold50290 | | pectin methylesterase inhibitor 1 | |  | | |  | | |  |
| C28548972 | | pectin methylesterase inhibitor 1 | |  | | |  | | |  |
| scaffold85568 | | pectin methylesterase PCR fragment F | | SLG5 | | | iDAFWA633 | | |  |
| C28739410 | | Pectinacetylesterase family protein | | SLG13 | | | DAFWA7025 | | |  |
| C27601111 | | Pectinacetylesterase family protein | |  | | |  | | |  |
| scaffold81045 | | Pectinacetylesterase family protein | | SLG12 | | | DAFWA4757 | | |  |
| scaffold1329 | | Pectinacetylesterase family protein | | SLG2 | | | DAFWA4291 | | |  |
| scaffold71004 | | Pectinacetylesterase family protein | |  | | |  | | |  |
| scaffold43493 | | Pectinacetylesterase family protein | | SLG6 | | | DAFWA6871 | | |  |
| scaffold1329 | | Pectinacetylesterase family protein | | SLG2 | | | DAFWA3575 | | |  |
| scaffold1186 | | Pectinacetylesterase family protein | |  | | |  | | |  |
| scaffold1329 | | Pectinacetylesterase family protein | | SLG2 | | | DAFWA3985 | | |  |
| scaffold43493 | | Pectinacetylesterase family protein | | SLG6 | | | DAFWA4660 | | |  |
| scaffold89845 | | Pectinacetylesterase family protein | | SLG9 | | | DAFWA3347 | | |  |
| scaffold92653 | | Pectinacetylesterase family protein | |  | | |  | | |  |
| scaffold97326 | | Pectinacetylesterase family protein | |  | | |  | | |  |
| scaffold87390 | | Pectinacetylesterase family protein | |  | | |  | | |  |
| scaffold53467 | | Pectinacetylesterase family protein | |  | | |  | | |  |
| scaffold28761 | | Pectinacetylesterase family protein | | SLG6 | | | DAFWA5444 | | |  |
| scaffold81390 | | Pectinacetylesterase family protein | |  | | |  | | |  |
| scaffold2931 | | Pectinacetylesterase family protein | |  | | |  | | |  |
| scaffold16815 | | Pectinacetylesterase family protein | |  | | |  | | |  |
| scaffold89111 | | Pectinacetylesterase family protein | | SLG6 | | | DAFWA3080 | | |  |
| scaffold60519 | | Pectinacetylesterase family protein | |  | | |  | | |  |
| scaffold92099 | | Pectinacetylesterase family protein | |  | | |  | | |  |
| scaffold89111 | | Pectinacetylesterase family protein | | SLG6 | | | DAFWA6090 | | |  |
| scaffold19203 | | Pectinacetylesterase family protein | |  | | |  | | |  |
| scaffold89111 | | Pectinacetylesterase family protein | | SLG6 | | | DAFWA4484 | | |  |
| scaffold69443 | | Pectinacetylesterase family protein | |  | | |  | | |  |
| scaffold83471 | | galacturonosyltransferase 1 | |  | | |  | | |  |
| scaffold71636 | | galacturonosyltransferase 1 | | SLG1 | | | DAFWA2590 | | |  |
| scaffold24236 | | galacturonosyltransferase 1 | |  | | |  | | |  |
| scaffold15767 | | galacturonosyltransferase 10 | |  | | |  | | |  |
| scaffold95610 | | galacturonosyltransferase 10 | |  | | |  | | |  |
| scaffold76263 | | galacturonosyltransferase 10 | |  | | |  | | |  |
| scaffold73221 | | galacturonosyltransferase 11 | |  | | |  | | |  |
| scaffold32853 | | galacturonosyltransferase 13 | |  | | |  | | |  |
| scaffold4579 | | galacturonosyltransferase 13 | | SLG8 | | | DAFWA7446 | | |  |
| scaffold92874 | | galacturonosyltransferase 13 | |  | | |  | | |  |
| scaffold32854 | | galacturonosyltransferase 13 | |  | | |  | | |  |
| scaffold91548 | | galacturonosyltransferase 13 | |  | | |  | | |  |
| scaffold64896 | | galacturonosyltransferase 15 | | SLG2 | | | DAFWA4927 | | |  |
| scaffold92190 | | galacturonosyltransferase 4 | |  | | |  | | |  |
| scaffold81673 | | galacturonosyltransferase 4 | | SLG14 | | | DAFWA6986 | | |  |
| scaffold77369 | | galacturonosyltransferase 4 | |  | | |  | | |  |
| scaffold1445 | | galacturonosyltransferase 6 | |  | | |  | | |  |
| scaffold82764 | | galacturonosyltransferase 7 | | SLG1 | | | DAFWA4465 | | |  |
| scaffold66386 | | galacturonosyltransferase 9 | |  | | |  | | |  |
| scaffold71955 | | galacturonosyltransferase-like 2 | |  | | |  | | |  |
| scaffold55099 | | galacturonosyltransferase-like 2 | |  | | |  | | |  |
| scaffold5722 | | galacturonosyltransferase-like 2 | |  | | |  | | |  |
| scaffold72048 | | galacturonosyltransferase-like 4 | |  | | |  | | |  |
| scaffold83440 | | galacturonosyltransferase-like 4 | |  | | |  | | |  |
| scaffold21301 | | galacturonosyltransferase-like 4 | | SLG15 | | | DAFWA3235 | | |  |
| scaffold21301 | | galacturonosyltransferase-like 4 | | SLG15 | | | DAFWA2951 | | |  |
